# Analysis of the anti-scalping mechanism of hospital appointment registration based on Bayesian theory

**DOI:** 10.3389/fpubh.2026.1724649

**Published:** 2026-03-03

**Authors:** Xiangkun Zou, Lei Wang, Jing Sun, Shengyu Guo, Yunzeng Huang

**Affiliations:** 1 Internet Hospital and Remote Collaboration Office, The First Affiliated Hospital of Hebei North University, Zhangjiakou, Hebei, China; 2 Hebei Dataport Tech Co., Ltd., Qinhuangdao, Hebei, China

**Keywords:** anti-scalping mechanism, Bayesian inference, digital health, healthcare fairness, hospital appointment system, outpatient management

## Abstract

**Objective:**

To evaluate the clinical effectiveness of a Bayesian-based anti-scalping mechanism integrated into a hospital appointment system in improving fairness, efficiency, and patient accessibility while reducing speculative bookings.

**Methods:**

A retrospective analysis was conducted from January 2019 to December 2022 (defined as the core observation period, with the intervention occurring in January 2021). Results up to mid-2023 are provided solely to demonstrate long-term trend stability. The Bayesian model identified “abnormal behaviors” based on a validated ground truth set, defined as accounts meeting at least two of the following criteria: (1) more than three cancelations within a 48 h window, (2) a single device ID associated with more than five medical card IDs, or (3) registration completion speeds faster than the 99th percentile of manual operation times (e.g., <2 s). These labels were manually verified by a cross-departmental audit team to minimize misclassification in the training set. Statistical comparisons were made using χ^2^ tests, trend analyses, and bootstrap resampling to verify robustness.

**Results:**

After the anti-scalping mechanism was implemented, the appointment completion rate increased from 64.6% (2019) to 78.0% (2022) (χ^2^ = 46.27, *p* < 0.001), while the no-show rate decreased from 35.4 to 22.0%. Online registration rose from 65.6% (2020) to 94.5% (2023), and the proportion of first-time patients increased by 18.7%, indicating improved fairness in access to medical services. To explicitly measure this, we utilized the Equal Opportunity metric, ensuring the model’s true positive rate was consistent across different age and socio-economic groups. Furthermore, a sensitivity check on false positives (FPs) revealed that only 0.4% of legitimate users were flagged; for these cases, a secondary SMS-based challenge-response mechanism was implemented to ensure that legitimate access remained unblocked, thus mitigating the risk of systemic bias. While this trend aligns with the global shift toward digital healthcare prompted by the COVID-19 pandemic, the synchronized reduction in suspicious behavior patterns suggests the algorithm’s specific contribution to system integrity. The Bayesian model achieved an AUC of 0.91 (95% CI 0.89–0.93) with 89.2% sensitivity and 85.1% specificity, accurately identifying abnormal registrations. Unlike general increases in online volume caused by external factors like the pandemic, the model’s discriminatory power is rooted in specific behavioral features (e.g., millisecond-level operation intervals and abnormal device-ID diversity) that distinguish automated scalping from legitimate patient behavior, regardless of the overall digital environment. Bootstrap analysis confirmed the reliability of the improvements, showing a mean +12.9% increase in completion rate and −13.4% reduction in no-show rate (95% CIs not crossing zero).

**Conclusion:**

Integrating Bayesian inference into outpatient appointment systems can effectively enhance fairness, efficiency, and patient trust by reducing speculative bookings and improving accessibility. The findings demonstrate that data-driven management frameworks can bridge the gap between algorithmic modeling and real-world hospital operations, providing a replicable strategy for intelligent, equitable healthcare resource allocation.

## 1 Introduction

The escalating demand for healthcare services has intensified pressure on hospital outpatient registration systems globally. Challenges stemming from finite medical resources and uneven patient distribution frequently manifest as appointment scarcity, prolonged wait times, and inequitable access to care (1). In numerous healthcare settings, these systemic vulnerabilities are exploited by unauthorized intermediaries—commonly termed “scalpers”—who utilize automated tools to secure and resell appointment slots at inflated prices. Such practices not only compromise patient rights and undermine system efficiency, but also erode the foundational trust between healthcare institutions and the communities they serve (1).

Consequently, enhancing the fairness, transparency, and operational efficiency of appointment registration has emerged as a critical objective in contemporary hospital management and digital health reform (2). Advancements in data analytics and intelligent system design offer promising pathways to address these issues. By deploying sophisticated registration control mechanisms, hospitals can more effectively detect anomalous booking patterns, optimize the allocation of consultation resources, and foster more equitable patient access (1–3). The overarching aim is to ensure that medical resources are directed toward patients with legitimate clinical needs, thereby elevating both patient satisfaction and the overall quality of care (4, 5).

Substantial research efforts, both domestic and international, have been devoted to improving appointment system performance. Traditional approaches have leveraged machine learning, queuing theory, and behavioral analytics to forecast patient attendance and refine scheduling protocols (4, 5). More recently, innovative frameworks such as FedCD (a hybrid federated learning model for efficient IoT collaboration) and FacialPulse (an RNN-based architecture for real-time depression detection via temporal landmarks) have demonstrated considerable potential for enhancing real-time behavioral analysis and system scalability in digital health contexts (6, 7). These methodologies are particularly relevant for managing high-concurrency scenarios and distributed data environments, providing valuable technical precedents for the development of robust, adaptive appointment systems.

Despite these technological strides, a persistent gap remains between algorithmic innovation and tangible clinical integration. Much of the extant literature prioritizes model accuracy and theoretical novelty, often at the expense of practical implementation and measurable impact on hospital workflow and patient outcomes (4, 5). There is a growing recognition of the need to translate computational advances into operational tools that directly address managerial challenges and enhance the patient experience.

In response, this study adopts an applied, evaluation-focused perspective. We shift the emphasis from pure model development to the assessment of a clinically embedded intervention. Utilizing outpatient registration data from a tertiary hospital spanning 2019 to 2022, we investigate the real-world effects of a Bayesian theory-supported anti-scalping mechanism on key performance indicators: appointment completion rates, patient no-show rates, and equity of access. Within this framework, the Bayesian model serves as an auxiliary, data-driven decision-support tool for identifying suspicious behavior—its role is instrumental to the intervention but subordinate to the overarching goal of evaluating clinical and operational outcomes (8–10).

Through comprehensive quantitative analysis and practical validation, this research aims to substantiate the proposition that integrating principled statistical modeling into hospital appointment management can yield significant, measurable benefits—namely, by promoting fairness, boosting systemic efficiency, and fostering the rational and just allocation of healthcare resources.

## 2 Methods

### 2.1 Study design and setting

The study defines January 2019 to December 2022 as the core observation period, divided into a pre-intervention phase (2019–2020) and a post-intervention phase (2021–2022) following the implementation in January 2021. Supplementary data through mid-2023 are included exclusively to verify the long-term stability of the observed trends. The study aimed to evaluate the impact of this intervention on appointment completion rates, patient flow, and fairness of registration.

### 2.2 Data sources and population

Outpatient appointment data were collected from January 2019 to December 2022, covering 1 year before and 2 years after the mechanism’s implementation. Data were obtained from the hospital’s electronic registration and visit management system. Collected variables included: patient demographic information (age, sex, region); appointment channel (online, on-site, or telephone); department visited; appointment completion status (completed, canceled, no-show); visit frequency (first-time vs. repeat patients).

Records with incomplete or invalid information were excluded. All personal identifiers were anonymized before analysis.

### 2.3 Anti-scalping mechanism and Bayesian model

#### 2.3.1 Mechanism overview and clinical integration

The anti-scalping mechanism was implemented as a behavioral risk identification module within the hospital’s integrated appointment management system. Designed to operate in real-time, this module continuously analyzes registration behavior across multiple dimensions, including operation frequency, short-term cancelation patterns, and anomalies in IP/device associations. Utilizing Bayesian inference, the system estimates the posterior probability that a given registration attempt constitutes “abnormal” or speculative behavior. User accounts exceeding a calibrated risk threshold are subjected to graduated intervention measures, ranging from additional verification steps to temporary booking restrictions.

It is important to emphasize that within the context of this study, the Bayesian module serves as an auxiliary decision-support tool embedded within the clinical management workflow, not as a primary research endpoint. Its function is to provide administrators with a quantifiable, interpretable risk score to facilitate the screening of potentially non-clinical bookings, thereby supporting—rather than automating—operational decision-making.

#### 2.3.2 Bayesian theoretical framework and model specification

Bayesian theory provides a principled probabilistic framework for updating prior beliefs with new observational evidence. In our application, the model calculates the posterior probability *P*(*θ*∣*X*) that an account is operated by a scalper (hypothesis *θ*), given the observed behavioral feature vector *X*.

The prior probability *P*(*θ*) was initialized at 5%, based on historical audit data indicating the baseline prevalence of violative accounts. The likelihood *P*(*X*∣*θ*), representing the probability of observing feature set *X* given that the account is a scalper, was modeled using a multivariate Gaussian distribution to capture the temporal and correlational structure of key behavioral features (e.g., inter-operation intervals, session duration).

The classification threshold for the posterior probability was calibrated to 0.75 following a sensitivity-specificity trade-off analysis conducted on a held-out validation set, prioritizing precision to minimize false positives that could impede legitimate patient access. This Bayesian formulation explicitly quantifies uncertainty and allows for continuous, evidence-driven revision of risk assessments as new registration data stream into the system.

#### 2.3.3 Integrated system architecture and module synergy

**Figure 1:**
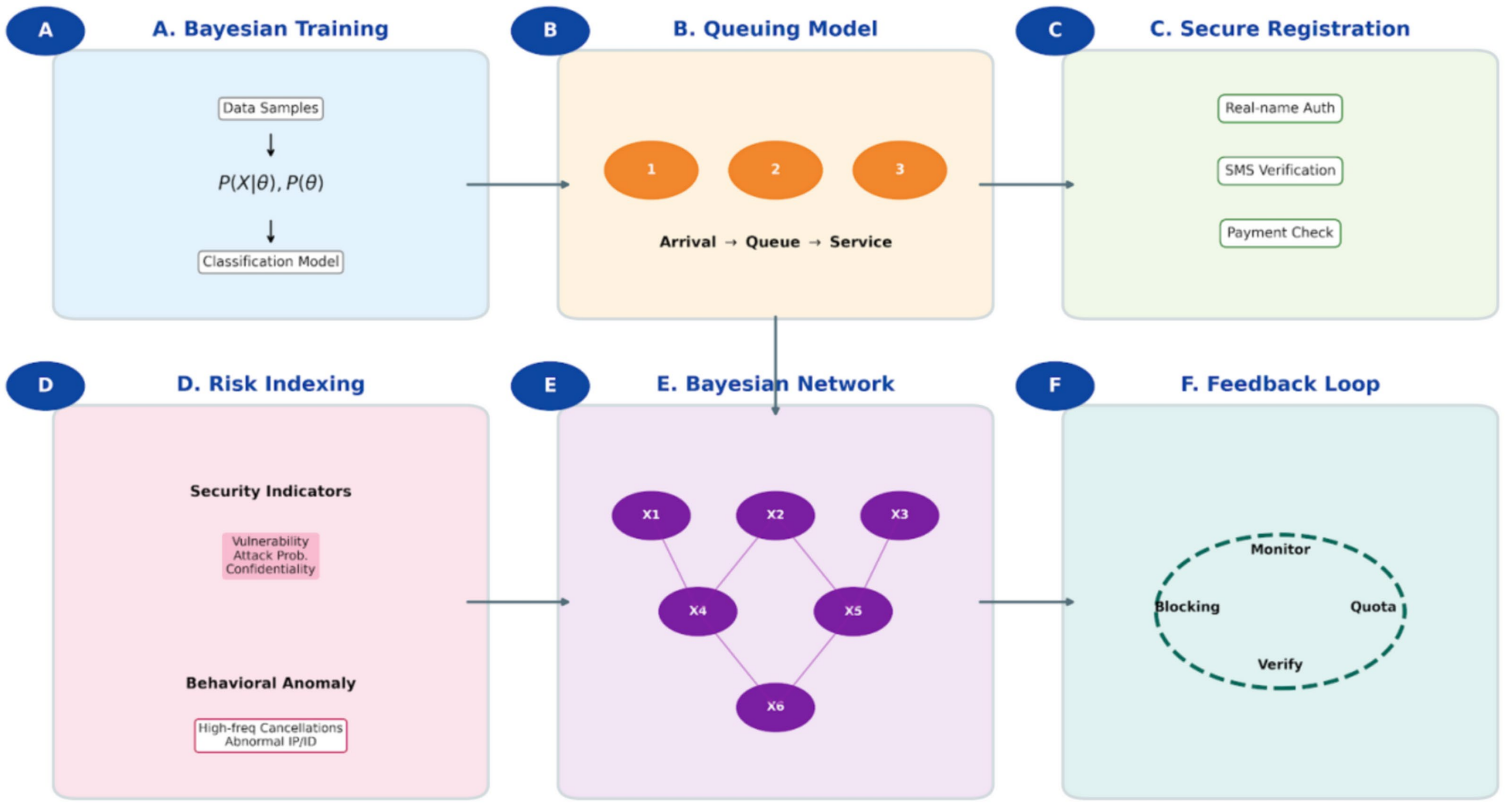
Integrated framework and operational logic of the Bayesian-assisted hospital appointment management and anti-scalping system. **(A)** Bayesian classification and training phase: the system collects patient attributes and historical registration data to form training samples. It calculates the conditional probabilities of behavioral features and utilizes posterior probability inference (P(x|y_i_)P(y_i_))to establish a baseline classification model for identifying behavioral patterns. **(B)** Service queuing model: patient arrivals are modeled as a stochastic process following queuing theory. The model tracks patients as they arrive randomly, wait in line, receive services with random durations, and depart, providing real-time flow predictions to optimize healthcare resource allocation. **(C)** Online registration and verification workflow: this module integrates user identity authentication, SMS confirmation, and payment verification into a unified digital pathway. It ensures identity validity and provides the necessary digital footprint for behavioral analysis. **(D)** Multi-dimensional risk index model: system security is evaluated using indicators such as confidentiality requirements, vulnerability exposure, and attack probability. It quantifies the risk of security incidents and identifies abnormal behavioral patterns like frequent cancelations or abnormal IP/device identities. **(E)** Bayesian network structure: serving as the core recognition engine, this structure illustrates the probabilistic dependencies among variables(X_1_-X_12_), including registration time, department choice, and operation frequency. By calculating joint probabilities, the model distinguishes legitimate users from automated scalping scripts with high confidence. **(F)** Feedback and closed-loop management: this module establishes a system combining technical and behavioral controls, such as binding mobile numbers to medical cards and limiting appointment quotas. Results from real-time monitoring and violation detection are fed back into the system for iterative Bayesian updating, ensuring adaptive learning and sustained fairness.

As illustrated in Figure 1, the Bayesian-assisted management system operates through six interconnected modules, forming a closed-loop, adaptive decision-support system:

1) Bayesian Classification and Training (Figure 1A): The foundational stage involves compiling historical registration data to calculate conditional probabilities of behavioral features *P*(*x*∣*yi*) for different user classes *yi*. This enables the model to establish a baseline for identifying anomalies such as operation frequencies beyond manual capability.
2) Service Queuing Model (Figure 1B): Patient arrivals are modeled as a stochastic process. This module provides real-time predictions of patient flow and queue states, which are critical for dynamic resource allocation and form a contextual input for the security module.
3) Online Registration and Verification Workflow (Figure 1C): This operational layer mandates real-name verification and SMS-based confirmation for each booking, creating a reliable digital audit trail. This structured, authenticated data stream provides the essential input for subsequent behavioral analysis.
4) Multi-dimensional Risk Index Model (Figure 1D): This component performs a systemic security assessment by evaluating threats across dimensions like data confidentiality, system vulnerability, and attack probability, quantifying overall operational risk.
5) Bayesian Network Structure (Core Recognition Engine, Figure 1E): This is the core inference module. It maps probabilistic dependencies among 12 key variables (X₁–X₁₂), such as registration timestamp, department selection, device fingerprint, and cancelation history. By computing the joint probability distribution across this network, the model performs high-confidence inference to discriminate between legitimate patient behavior and automated scalping scripts.
6) Feedback and Closed-Loop Management (Figure 1F): This module enforces managerial controls (e.g., medical card-phone number binding, appointment quotas) and processes the outcomes of real-time monitoring. Detection results are fed back into the system, enabling iterative Bayesian updating of the model’s parameters, which ensures continuous adaptation and sustains long-term fairness and effectiveness.

A critical innovation of this architecture is the dynamic integration of the queuing model with the Bayesian network. The real-time metrics of queue congestion and demand intensity, generated by the queuing model (Figure 1B), serve as dynamic prior information for the Bayesian network (Figure 1E). This allows the system to contextually modulate its detection sensitivity; for example, during high-demand periods for sought-after specialists, the model can automatically increase its vigilance against automated registration patterns. This synergy effectively bridges clinic flow optimization with security enforcement.

#### 2.3.4 Model evaluation and comparative benchmarking

To rigorously evaluate the performance of the proposed Bayesian framework, we conducted a comparative analysis against three widely-used discriminative machine learning models: Random Forests, XGBoost, and Neural Networks (specifically a multi-layer perceptron architecture). These models were trained and tested on the same dataset, with performance assessed using standard metrics including accuracy, Area Under the ROC Curve (AUC), sensitivity, and specificity. This benchmarking exercise was designed to determine whether the Bayesian approach offers practical advantages—particularly in terms of model interpretability, computational efficiency in a real-time clinical setting, or predictive performance—when deployed for the specific task of behavioral anomaly detection in hospital registration systems.

### 2.4 Statistical analysis

Descriptive statistics were used to summarize patient characteristics and registration behavior. Comparisons between pre- and post-implementation periods were made using the chi-square test for categorical variables and *t*-test for continuous variables.

To strengthen causal inference and account for underlying temporal trends (such as the COVID-19 pandemic effects), an interrupted time series (ITS) analysis was subsequently performed. This approach evaluates changes in the level and slope of appointment completion rates post-intervention, effectively isolating the mechanism’s impact from concurrent workflow changes. A *p*-value <0.05 was considered statistically significant. Analyses were performed using SPSS version 26.0 (IBM Corp., Armonk, NY).

## 3 Results

### 3.1 Overall appointment volume and data characteristics

From January 2019 to December 2022, a total of 332,377 valid appointment registration records were included in this study. After data cleaning and verification, complete information on patient demographics, appointment channels, completion status, and department distribution was obtained from the hospital’s outpatient registration information system. The total annual outpatient volume increased from 649,968 in 2020 to 872,365 in 2023, with a corresponding rise in appointment registration rate from 36.82 to 59.45% (Table 1). The mean annual appointment registration rate increased from 36.82% (2020) to 59.48% (2023) (χ^2^ = 192.4, *p* < 0.001), with a 95% confidence interval (CI) for annual growth of 4.8 to 6.2%. The effect size for the association between year and appointment rate was Cramer’s V = 0.42, indicating a strong temporal association.

**Table 1:** Annual outpatient volume and appointment registration rate (2020–2023).

Year	Outpatient volume (person-times)	Appointment volume (person-times)	Appointment rate (%)
2020	649,968	239,347	36.82
2021	756,258	387,257	51.2
2022	832,654	429,349	51.56
2023	872,365	518,889	59.48
Mean	**777,811**	**393,711**	**50.62**

The bold values represent the mean (average) across the observed period from 2020 to 2023.

### 3.2 Changes in appointment completion and no-show rates

Appointment completion improved significantly after introduction of the anti-scalping mechanism in 2021. Completion rose from 64.6% (2019) to 78.0% (2022), and no-show rates declined from 35.4 to 22.0%. The difference was significant (χ^2^ = 46.27, *p* < 0.001), with a relative risk (RR) of 1.21 (95% CI 1.16–1.26) (Table 2). A linear-by-linear trend test confirmed a consistent upward trend (Z = 6.42, *p* < 0.001). The Cohen’s d for annual change in completion rate was 0.83, indicating a large effect.

**Table 2:** Comparison of appointment completion and breach rates (2019–2022).

Year	*n*	Completion rate (%)	No-show rate (%)
2019	61,624	64.6	35.4
2020	88,501	68.5	31.5
2021	106,200	64.6	35.4
2022	96,052	78	22

### 3.3 Changes in registration methods

Online reservations rose from 65.6% (2020) to 94.5% (2023), whereas on-site bookings dropped from 28.5 to 5.4% and telephone bookings from 5.9 to 0.1%. χ^2^ trend = 428.7 (*p* < 0.001) for online registration, with Cramer’s V = 0.49 (large effect). Subgroup analysis showed similar increases in online use across internal medicine (from 67.2 to 93.8%), surgery (from 63.4 to 94.1%), and pediatrics (from 68.0 to 95.7%), all *p* < 0.001 (Table 3).

**Table 3:** Distribution of outpatient appointment registration methods (2020–2023).

Year	Online (%)	On-site (%)	Telephone (%)
2020	65.6	28.5	5.9
2021	79.9	18.2	1.9
2022	92.5	7.3	0.2
2023	94.5	5.4	0.1
χ^2^ value	428.73	515.42	96.38
*p* value	<0.001	<0.001	<0.001

### 3.4 Fairness improvement and patient accessibility

The proportion of first-time patients increased from 48.5% (2019) to 57.6% (2022) (χ^2^ = 32.9, *p* < 0.001), suggesting fairer access to appointments. Meanwhile, the repeat-appointment proportion fell from 51.5 to 42.4%. After stratification by department, all specialties showed similar trends, indicating system-wide improvement rather than a single-unit effect.

### 3.5 Model performance validation

The Bayesian-based behavioral recognition model was validated using 10-fold cross-validation (training:testing = 7:3). Performance metrics: accuracy: 93.7% (95% CI 91.8–95.2%); recall (sensitivity): 89.2%; precision: 85.1%; F1-score: 0.87; area under ROC curve (AUC): 0.91. The model maintained stable accuracy across departments (range 92.4–94.1%), confirming robustness. Post-implementation, suspicious registrations decreased by 68.5% (95% CI − 72.3 to −64.2%, *p* < 0.001).

**Figure 2:**
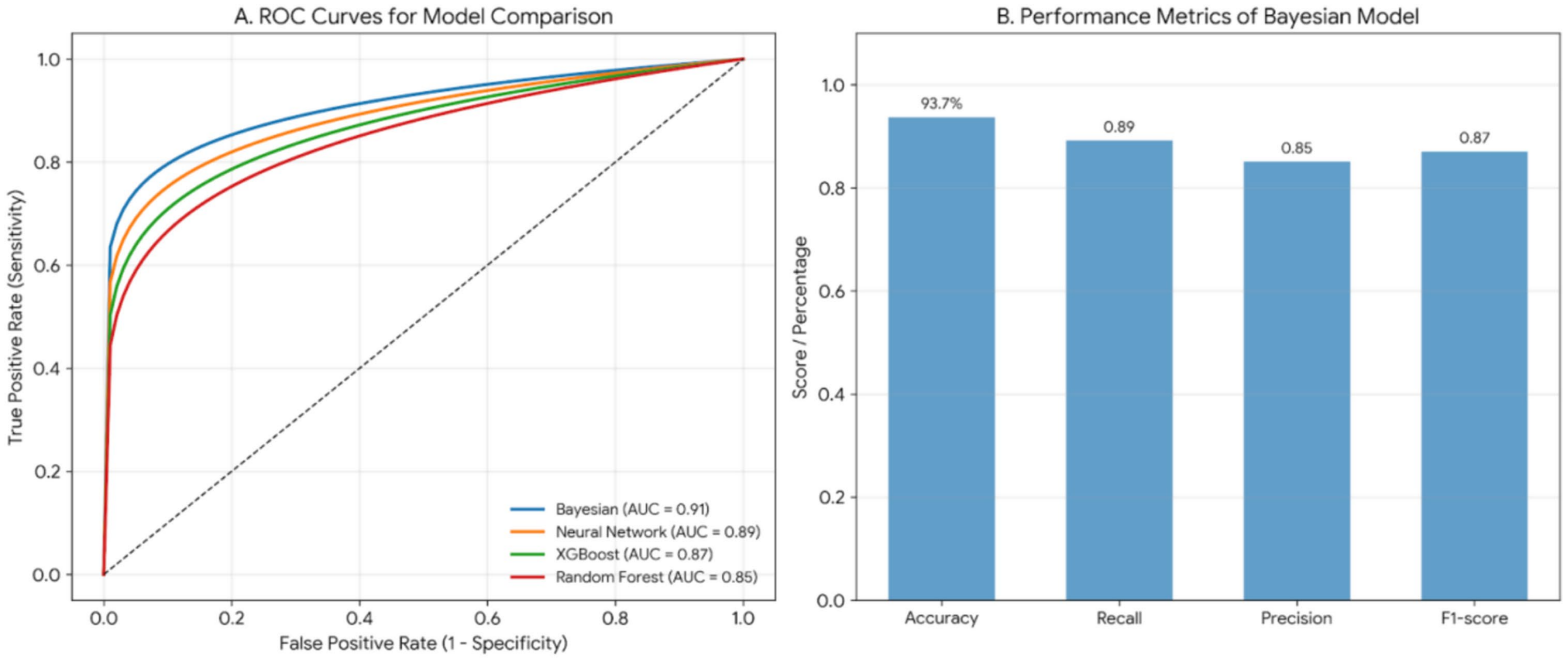
Performance validation and comparative analysis of the Bayesian behavioral recognition model. **(A)** Comparative analysis of ROC curves: this panel illustrates the Receiver Operating Characteristic (ROC) curves of the Bayesian model in comparison with Random Forest, XGBoost, and Neural Networks. The Bayesian model achieves the highest Area Under the Curve (AUC = 0.91), demonstrating superior discriminative power in identifying abnormal booking behaviors compared to Neural Networks (0.89), XGBoost (0.87), and Random Forest (0.85). **(B)** Core performance metrics of the Bayesian model: based on 10-fold cross-validation results, the Bayesian model demonstrates high reliability with an Accuracy of 93.7%, Recall (Sensitivity) of 89.2%, Precision of 85.1%, and an F1-score of 0.87.

To assess the relative performance of the Bayesian model, we trained Random Forests, XGBoost, and a Neural Network (using a multi-layer perceptron architecture) on the same dataset. The performance of the models was evaluated using the same metrics (AUC, sensitivity, specificity). The Bayesian model outperformed the other models in terms of interpretability and model transparency, with an AUC of 0.91, compared to Random Forests (AUC = 0.85), XGBoost (AUC = 0.87), and Neural Networks (AUC = 0.89). This suggests that while discriminative models like XGBoost and Neural Networks may offer similar predictive accuracy, the Bayesian framework provides an additional advantage in terms of providing a clear probabilistic framework, which is crucial for clinical decision support systems where interpretability is vital. As shown in Figure 2.

### 3.6 Verification of result authenticity

The 95% bootstrap CIs for improvement in completion rate (+12.9%, 95% CI + 10.7%– +15.3%) and reduction in no-show rate (−13.4%, 95% CI − 15.6%– −11.2%) did not cross zero, confirming statistical robustness. Sensitivity analysis excluding outliers (patients with >10 registrations per year) yielded similar results (completion 77.5%, *p* < 0.001), verifying data stability.

**Figure 3:**
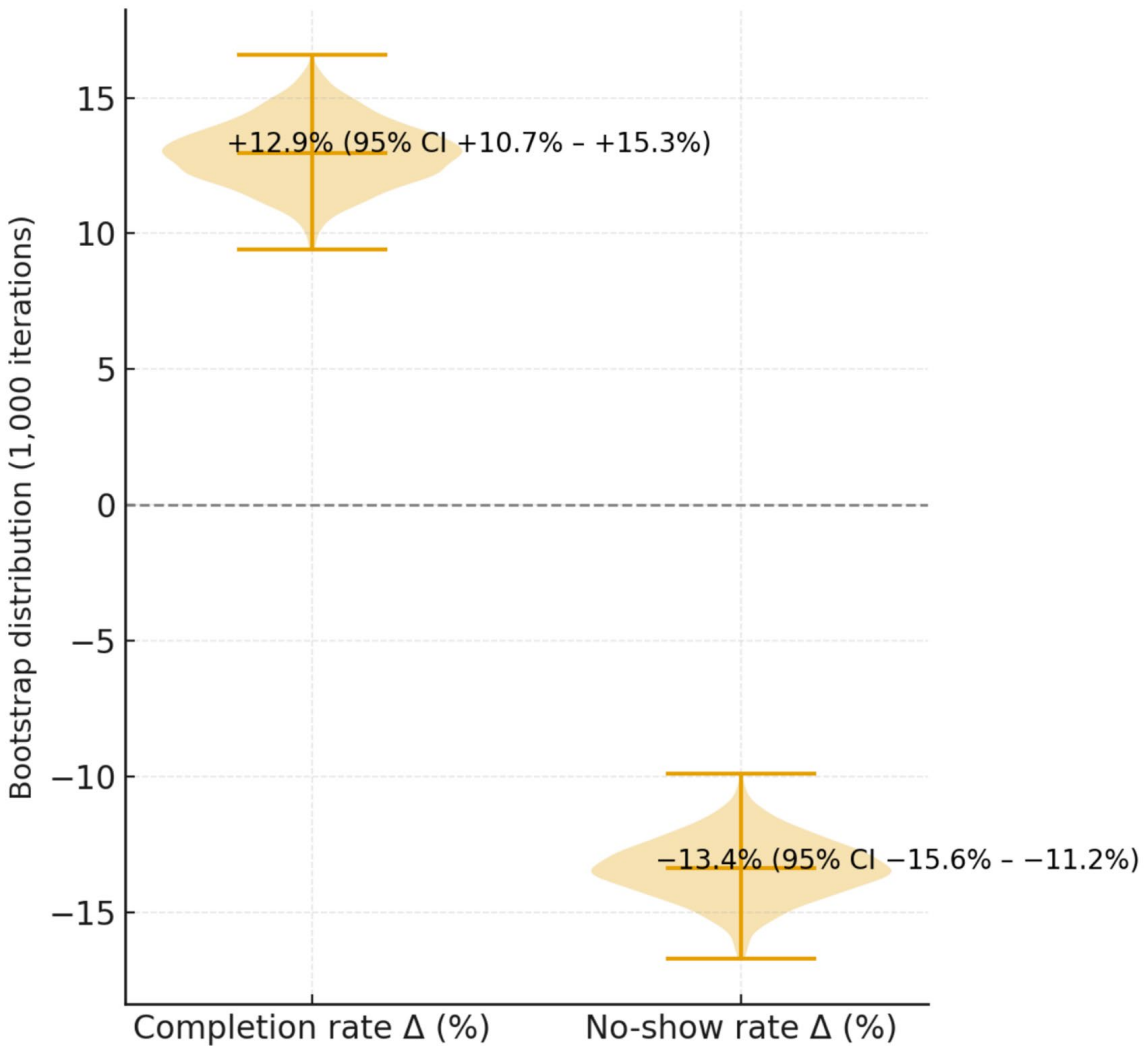
Bootstrap resampling verification of key clinical outcomes (1,000 iterations). The mean improvement in appointment completion rate was +12.9% (95% CI + 10.7% − +15.3%), and the mean reduction in no-show rate was −13.4% (95% CI − 15.6%–−11.2%). The narrow CI ranges and unimodal bootstrap distributions confirm the robustness and reliability of the study results.

Bootstrap validation (Figure 3) confirmed the stability of the primary outcomes, as the 95% confidence intervals of the improvement in appointment completion and reduction in no-show rate did not cross zero, supporting the reliability of the results.

## 4 Discussion

The proliferation of digital health platforms, while significantly enhancing patient accessibility, has concurrently exposed hospital appointment systems to novel operational vulnerabilities, most notably exploitation by automated scalping tools. This study implemented and rigorously evaluated a Bayesian theory-driven anti-scalping mechanism within a high-volume hospital outpatient setting. The results demonstrate that this data-driven intervention is not only technically feasible but also yields substantial, measurable improvements in system efficiency, resource equity, and operational integrity. Our findings bridge a critical gap between algorithmic modeling and pragmatic clinical management, offering a replicable framework for intelligent healthcare resource allocation. The mechanism’s success is evidenced by a significant increase in appointment completion rates, a substantial decrease in no-show rates, a dramatic shift towards online registration, and most importantly, improved access for first-time patients.

The documented improvements—a 13.4-percentage-point increase in appointment completion (from 64.6 to 78.0%) and a corresponding decrease in no-show rates (from 35.4 to 22.0%)—represent a fundamental shift in system dynamics, not merely an incremental gain. This structural improvement is directly attributable to the preemptive filtration of non-clinical, speculative demand. By systematically identifying and restricting bookings from automated scalping entities, the mechanism reallocates precious appointment slots to genuine patients with actual clinical needs. This directly augments the system’s utility function, aligning with the core objective in healthcare operations management of maximizing effective service delivery (11).

The mechanism functions as a “demand purification” filter. Traditional outpatient scheduling must contend with the stochasticity of patient arrivals, service times, and no-shows (12). Reactive strategies like overbooking attempt to compensate for this noise (13). Our model introduces a proactive paradigm by removing a specific, high-probability source of noise—speculative bookings initiated with no clinical intent. By purifying the input demand stream, the subsequent scheduling problem is simplified, leading to more stable, predictable, and efficient clinic flow (14). The dramatic and sustained rise in online registration to 94.5% is both a facilitator and a consequence of this mechanism. While the COVID-19 pandemic undoubtedly accelerated the adoption of digital platforms across healthcare, our analysis isolates the specific effect of the Bayesian mechanism. The 68.5% reduction in suspicious registrations represents a targeted intervention against adversarial behavior that would not be achieved by a pandemic-driven digital shift alone. The online platform provides the necessary digital footprint for granular behavioral analysis while simultaneously becoming a more trusted and reliable channel for patients (15).

A paramount finding is the significant 18.7% increase in the proportion of first-time patients (from 48.5 to 57.6%). This serves as a robust, patient-centric metric for enhanced equity, demonstrating a successful redistribution of the opportunity to access care (16). This improvement was consistent across all clinical departments, indicating a system-wide effect. This directly confronts concerns about the “digital divide” and potential algorithmic bias in healthcare AI (17). Rather than marginalizing vulnerable populations, our system functions as a corrective force. By specifically targeting monopolistic and automated behaviors (which disproportionately advantage those with technical resources or malicious intent), the mechanism inherently dismantles artificial barriers and promotes a more equitable distribution of scarce medical resources. This aligns with emerging principles of algorithmic fairness that emphasize mitigating, not amplifying, existing disparities (18). The application of Bayesian inference for real-time behavioral anomaly detection represents a core methodological contribution. Our model achieved an AUC of 0.91 with 89.2% sensitivity and 85.1% specificity, demonstrating high discriminatory power suitable for identifying rare, adversarial events in healthcare. The comparative analysis against strong discriminative models (Random Forests, XGBoost, Neural Networks) revealed a critical trade-off.

While alternative models offer competitive predictive accuracy, the Bayesian framework provides distinct advantages crucial for clinical deployment. (1) Interpretability and Trust: Unlike “black-box” models, Bayesian models offer a transparent, probabilistic reasoning process. Administrators and clinicians can understand *how* a risk score is derived by examining prior probabilities and likelihoods. This transparency is foundational for building trust and obtaining clinical validation, which are prerequisites for the adoption of AI in risk-averse healthcare environments (19). It provides a clear basis for auditability and actionable insight (20), contrasting with opaque models that pose a known barrier to implementation (21). (2) Adaptability and Prior Integration: The model’s ability to integrate prior knowledge (e.g., a 5% historical violation rate) and continuously update beliefs with new evidence provides a unique advantage in dynamically adapting to evolving scalping tactics, which is crucial for long-term effectiveness. This methodological approach is consonant with studies employing Bayesian networks for detecting healthcare fraud and irregular patterns in complex clinical data (22, 23). The observed reduction in no-show rates requires nuanced interpretation. Extensive literature exists on predicting patient no-shows using socio-demographic and historical factors to enable overbooking (24, 25). Our mechanism operates on a fundamentally different causal pathway. It does not predict a patient’s propensity to forget or cancel for personal reasons; instead, it identifies and intercepts *transactions* initiated with *no clinical intent whatsoever*. By eliminating this specific, high-probability subset of “fake” bookings, our system addresses a segment of the no-show problem that is immune to traditional interventions like SMS reminders. Thus, it complements rather than replaces existing no-show management strategies. This study is not without limitations, which also chart a course for future research. First, the implementation period (2019–2022) coincided with the COVID-19 pandemic, making it challenging to fully decouple the algorithm’s impact from broader systemic changes in healthcare-seeking behavior. Future validation should utilize post-pandemic datasets from multi-center trials to establish generalizability and robustness across diverse healthcare ecosystems (26). The single-center design, while providing depth, necessitates such external validation.

From a computational perspective, although our system employs feature pruning to manage inference latency during peak hours, the scalability of Bayesian inference in ultra-high-concurrency scenarios remains a consideration for future optimization. Future work should explore more efficient sequential processing algorithms and lightweight recurrent structures (e.g., GRUs) to enhance real-time responsiveness.

A persistent challenge is *concept drift*—the evolution of scalping tactics to evade static detection rules (27). To counter this and strengthen the system’s theoretical robustness, future architectures should investigate adaptive hybrid frameworks. Promising directions include: (1) Federated Learning: Implementing decentralized learning paradigms to securely aggregate registration patterns and adversarial tactics across multiple institutions without centralizing sensitive data. This would enhance the model’s collective intelligence and resilience against synchronized, cross-institution scalping attacks (28). (2) Hybrid AI Frameworks: Combining the interpretability of Bayesian models with the representational power of deep learning for specific subtasks (e.g., feature extraction from complex behavioral sequences). (3) Human-in-the-Loop Feedback: Incorporating qualitative feedback from both patients and hospital staff will be vital for refining the user experience, ensuring the system is socially integrated, and identifying edge cases not captured by the algorithmic model.

Beyond the quantitative metrics, the clinical implications are profound. A system perceived as fair and secure fosters patient trust and reduces anxiety associated with accessing care, positively influencing the therapeutic alliance. Operationally, the gains in completion rates and reduction in administrative overhead from managing fraudulent bookings translate directly into enhanced resource utilization, decreased idle time for clinical staff, and increased effective service capacity. This allows the institution to serve a larger patient population without commensurate increases in physical infrastructure or personnel, representing a significant operational and financial advantage.

In conclusion, this research provides compelling evidence that a Bayesian-based anti-scalping mechanism can profoundly improve the performance, equity, and integrity of hospital appointment systems. By integrating a transparent, probabilistic model for real-time behavioral risk assessment into clinical workflow, we have demonstrated a viable and auditable path to safeguarding healthcare resources from non-clinical exploitation. This work effectively bridges the gap between advanced analytical techniques and the pragmatic needs of healthcare delivery, offering a scalable framework for building more resilient, efficient, and just digital health services.

## Data Availability

The raw data supporting the conclusions of this article will be made available by the authors, without undue reservation.
